# Utilization of Avocado and Mango Fruit Wastes in Multi-Nutrient Blocks for Goats Feeding: In Vitro Evaluation

**DOI:** 10.3390/ani10122279

**Published:** 2020-12-03

**Authors:** Carlos Navarro Marcos, María Dolores Carro, Julia E. Fernández-Yepes, Lesly Arbesu, Eduarda Molina-Alcaide

**Affiliations:** 1 Departamento de Producción Agraria, ETSIAAB, Universidad Politécnica de Madrid, Ciudad Universitaria, 28040 Madrid, Spain; navarro-88@hotmail.com (C.N.M.); mariadolores.carro@upm.es (M.D.C.); 2 Estación Experimental del Zaidín (Consejo Superior de Investigaciones Científicas), Profesor Albareda 1, 18008 Granada, Spain; julia.fernandez@eez.csic.es (J.E.F.-Y.); leslyarbesu@gmail.com (L.A.)

**Keywords:** peels, pulp, tropical fruits, preserving methods, ruminal fermentation, methane

## Abstract

**Simple Summary:**

The demand for animal products generated with high animal welfare standards and low environmental impact is continuously increasing. Moreover, the growing awareness of consumers about the importance of a healthy diet to reduce the prevalence of dietary illnesses has increased the consumption of vegetables and fruits, generating more vegetable wastes. Using these wastes in animal feeding would reduce the pollution caused by their accumulation, but their nutritive value needs to be assessed. We analyzed the chemical composition and in vitro ruminal fermentation of avocado and mango fruit wastes (peels and a pulp:peels (PP) mixture), and the potential of including the PP mixture into multi-nutrient blocks (MB) for goats feeding. Tested wastes had high-moisture content, but whereas those from mango were rich in non-structural carbohydrates, those from avocado had high fat content. Mango wastes were fermented at a greater extent and faster rate than avocado ones. Only subtle differences were observed in the fermentation of MB including PP from either avocado or mango. Using the PP mixture in MB for goats seems to be a viable solution to reduce the waste’s environmental impact, but studies assessing the MB acceptance by the animals and their stability over long-time storage periods are needed.

**Abstract:**

This study was conducted to investigate the nutritive value of avocado and mango fruit wastes, and to assess the possibility of preserving the wastes into multi-nutrient blocks (MB). Both peels and a pulp:peels (PP) mixture of each fruit were analyzed for chemical composition and in vitro fermentation with goats’ ruminal fluid. Wastes had low-dry matter (DM) content (<250 g/kg), with those from mango having high non-structural carbohydrates content (>800 g/kg DM) and those from avocado high fat levels (>580 g/kg DM). Mango wastes were fermented at a greater extent and faster rate than avocado ones. The PP mixture of each fruit was included into multi-nutrient blocks (MB) formulated to have similar chemical composition. There were only subtle differences in the fermentation of MB including wastes from either avocado or mango, but fermentation of avocado-MB resulted in significantly (*p* ≤ 0.032) greater acetate and lower propionate proportions than mango-MB. Including the PP mixture in the formulation of MB for goats feeding is a feasible option to reduce the environmental impact of avocado and mango fruit wastes, but studies on the acceptance of the MB by goats and their stability over long-time storage periods are needed.

## 1. Introduction

The demand for tropical fruits, such as avocado and mango, has increased over the last years [1] because they exhibit a wide range of bioactive compounds beneficial for human health. Avocado is a tropical crop with more than 6 million tons produced annually [2], and its consumption is rapidly growing worldwide, especially in Europe [3]. Although much of avocado production is consumed fresh, many avocado food (drinks, guacamole, sauces, etc.) and cosmetic (shampoo, skin creams, oils, etc.) products are also available in the market. Avocado cultivation and processing generate large quantities of waste products, mainly peels and kernels, which represent between 18% and 13% of fruit fresh weight [4]. Mango is considered the most important tropical crop cultivated worldwide, and currently more than 46 million tons are produced annually [2], with high expectations of increasing production due to the increased consumer demand [5]. Mango fruits are consumed fresh, but they can also be processed (canned, juices and nectar, dehydrated fruit, frozen, etc.), and these processes generate high amounts of wastes, which are mainly composed of peels and kernels that can account for 35–60% of fruit fresh weight [5]. In the processing of both fruits, pulp is also originated as waste.

Utilization of agricultural wastes in animal feeding is a viable option to reduce the environmental impact associated to their management and to decrease the cost of animal feeding. In addition, including avocado and mango fruit wastes in animal diets could produce beneficial effects to the animals due to their bioactive compounds, such as polyphenols and tannins [4,6]. However, these wastes have high-moisture content, being highly perishable and prone to rapid spoilage [7]. Furthermore, the generation of these wastes is concentrated in the harvesting period. All these factors make the use of these wastes in animal feeding difficult, and low-cost preservation methods are required [7]. An interesting cost-effective preservation method, but still not widely used, is the inclusion of fruit wastes in multi-nutrient blocks (MB) [8], which are a solid mixture of feeds, urea, binder, salt and mineral and vitamin supplements [9]. The effects of feeding MB including avocado wastes to goats on milk yield and composition have been recently studied [10], but to our best knowledge, there is no information on the possibility of including mango wastes in MB. However, several studies have investigated the direct feeding of mango wastes to ruminants [11,12,13] and non-ruminants [14,15], indicating that they can be used as alternative feeds [16]. The aim of this study was therefore to assess the chemical composition and in vitro ruminal fermentation of two type of wastes from avocado and mango, peels and a mixture of pulp and peels (PP), and to analyze the in vitro ruminal fermentation of MB including PP wastes of each fruit.

## 2. Materials and Methods

### 2.1. Avocado and Mango Fruit Wastes

Avocado and mango fruits were collected from a local market in Granada (Spain). Fruits were first cleaned and carefully peeled. The peels were then cleaned of any flesh. The kernels from all fruits were extracted and they were discarded after the rest of the pulp was removed and mixed. The pulp of the fruit was mixed with that obtained from cleaning the peels and kernels and homogenized using an industrial mixer (SAMMIC S.L., Azkoitia, Spain). All parts of the fruits (peel, kernel and pulp) were weighed to state their relative proportions in the fruit. A portion of the pulp and peels were mixed up (on a weight basis) at a ratio of 81:19 for avocado and 65:35 for mango, representing their proportions in the fruit. All samples were stored frozen until analysis.

### 2.2. Animals and Feeding

All animals used in this study were cared for and handled in accordance with the Spanish guidelines for experimental animal protection (Royal Decree 53/2013 on the protection of animals used for experimentation or other scientific purposes). Procedures for rumen content sampling were approved by the Ethic Committee for Animal Experimentation of the Spanish Research Council and Junta de Andalucía (Approval number 22/06/2016/115). Three adult, dry, non-pregnant, rumen-fistulated Murciano-Granadina goats (46.9 ± 2.15 kg body weight) were housed individually and used as inoculum donors for the in vitro incubations. The internal diameter of the cannula was 4.5 cm, which allows the collection of both liquid and solid fractions from different parts of the rumen. Goats were fed alfalfa hay twice a day at 9:00 and 18:00 h to meet energy maintenance requirements [17] and had free access to fresh water.

### 2.3. Experimental Design

This study comprised two different experiments in which the same methodology was used. The chemical composition and in vitro rumen fermentation of avocado and mango wastes was studied in Experiment 1, whereas Experiment 2 assessed the chemical composition and in vitro rumen fermentation of multi-nutrient blocks (MB) including either avocado (AMB) or mango wastes (MMB) and of mixed diets including MB and alfalfa hay. In both experiments, all feed samples were ground (1 mm screen) before chemical analysis and in vitro incubations.

#### 2.3.1. Experiment 1. In Vitro Fermentation of Avocado and Mango Fruit Wastes

Batch cultures of ruminal microorganisms were used to assess the in vitro ruminal fermentation, CH_4_ production and kinetic of gas production of avocado and mango peels and a mixture of pulp and peels in a 81:19 and 65:35 ratio respectively, representing the proportions of each part in the fruit. The experimental procedure has been described in detail by Molina-Alcaide et al. [18]. Briefly, rumen content from each of the three goats was collected before the morning feeding, pooled, and immediately taken to the laboratory into thermal flasks. Rumen contents were strained through four layers of cheesecloth, and mixed (1:4 ratio) with the buffer solution described by Goering and Van Soest [19] (without adding trypticase). The procedure was conducted at 39 °C under continuous flushing with CO_2_.

Three identical 144 h incubation runs were carried out, and in each of them, 4 bottles per sample and 4 bottles without substrate (blanks) were used. In addition, two conventional feeds widely used in ruminant feeding (wheat middlings and corn grains) were included in the incubations to serve as reference. Four hundred mg of each sample were carefully weighed into 120 mL bottles before adding 48 mL of the buffered rumen fluid into each bottle. Bottles were sealed with rubber stoppers and aluminum caps, and incubated at 39 °C in a water bath. Two bottles for each sample and two blanks were used to assess the gas production kinetics of the samples. Both the pressure inside the bottle and the volume of gas produced were measured at 2, 4, 6, 8, 12, 24, 48, 72, 96, 120 and 144 h of incubation using a Wide-Range Pressure Meter (Sper Scientific LTD, Scottsdale, AZ, USA) and a glass-calibrated syringe (Ruthe^®^, Normax, Marinha Grande, Portugal). At the end of the incubation period, fermentation was stopped by placing the bottles in iced-water. The content of the bottles was frozen, freeze-dried and analyzed for neutral detergent fiber (NDF) to calculate the true dry matter (DM) degradability (TDMD) [20].

The rest of the bottles (2 per sample and 2 blanks) were incubated for 24 h before measuring the volume of gas produced as described above. A gas sample (10 mL) was stored in an evacuated tube (Terumo Europe N.V., Leuven, Belgium) for analysis of CH_4_ concentration. The bottles were then uncapped, the pH was measured immediately (Crison Basic 20 pH-meter, Crisson Instruments, Barcelona, Spain) and the bottles were placed in iced-water. The following samples were then taken: 2 mL were mixed with 2 mL of deproteinizing solution (20 g of metaphosphoric acid and 0.6 g of crotonic acid per l) for volatile fatty acids (VFA) analysis, and 1 mL was preserved with 1 mL of 0.5 M HCl for NH_3_-N analysis. Samples were immediately frozen (−20 °C).

#### 2.3.2. Experiment 2. In Vitro Fermentation of Multi-Nutrient Blocks (MB) Including Avocado and Mango Fruit Wastes and of Diets Based on Alfalfa Hay and MB

Two types of MB including the PP mixture, from either avocado or mango, used in Experiment 1 (AMB and MMB, respectively), were formulated to have similar nitrogen (N) and neutral detergent fiber (NDF) content. The feed ingredient composition of the MB is shown in Table 1. The MB were prepared following the methodology described by Molina-Alcaide et al. [8]. Briefly, all feed ingredients were thoroughly mixed and water was added before packing and hard-pressing the mixture into metal round molds. Finally, the MB were removed from the molds and air-dried outdoors before analysis of chemical composition and in vitro rumen fermentation. Additionally, 50:50 mixtures of alfalfa hay and either AMB or MMB (on fresh weight basis) were prepared to resemble mixed diets used for goats feeding in practical conditions.

Incubation procedures were as described in Experiment 1, and AMB, MMB, alfalfa hay:AMB and alfalfa hay:MMB mixtures at a 50:50 ratio were incubated. Alfalfa hay was also included as a reference feed. As in Experiment 1, three incubation runs were carried out using 4 bottles per sample, and 4 blanks in each of them. Sampling was as described in Experiment 1 for both 144 and 24 h incubations.

### 2.4. Chemical Analyses

Ground samples (1 mm) were analyzed for dry matter (ID 934.01), ash (ID 048.13) and ether extract (EE; ID 920.39) according to the methods of the Association of Official Analytical Chemists [21]. Analyses of total N, NDF, acid detergent fiber (ADF) and lignin were performed for samples of mango wastes and for defatted avocado wastes. Total N analysis was performed using a Leco TruSpec CN^®^ (Leco Corporation, St. Joseph, MI, USA). The NDF, ADF and lignin were analyzed according to Van Soest et al. [22] using a Fiber Analyzer (ANKOM Model 220, ANKOM Technology Corporation, Fairport, NY, USA) and α-amylase for NDF analyses. Both NDF and ADF contents were expressed exclusive of residual ash and without sulfite. An adiabatic calorimeter (Model 1356, Parr Instruments Co., Moline, IL, USA) was used to assess the energy content of the samples, and their content in non-structural carbohydrates (NSC) were calculated as (organic matter (OM)–crude protein–NDF–EE).

The CH_4_ concentration in the gas produced was determined by gas chromatography using a Hewlett HP 5890 Series II gas chromatograph (Hewlett Packard, Waldbronn, Germany) equipped with a flame ionization detector and a HP-INNOWAX crosslinked polyethylene glycol column (25 m × 0.2 mm × 0.2 µm) and using He as carrier gas. A sample of 0.5 mL of the gas obtained from the bottles was injected using a 1 mL Hamilton gastight sample-lock^®^ syringe (Hamilton, NV, USA). Total and individual volatile fatty acids (VFA) were analyzed by gas chromatography following the methodology described by Isaac et al. [23], and NH_3_-N concentrations were determined by a colorimetric method [24].

### 2.5. Calculations and Statistical Analyses

The amount of gas produced in the blank bottles was used to correct the gas production values for the gas release from endogenous materials. The NLIN) procedure of SAS Institute [25] was used to adjust the gas values to the model of Krishnamoorthy et al. [26]: y = PGP (1 − e^(−*c*(t − *lag*))^), where y is gas (mL) measured at a sampling time t (h), PGP is the potential gas production (asymptote; mL), *c* is the fractional gas production rate (%/h) and *lag* (h) is the delay in starting gas production. The gas production rate between the start of the incubation and the time at which 50% of PGP is reached (average gas production rate; AGPR; ml/h) was estimated as suggested by France et al. [27]: AGPR = PGP *c*/[2(ln2 + *c lag*)]. Finally, the DM effective degradability (DMED) was estimated using the equation proposed by France et al. [27]: DMED = [(TDMD *c*)/(*c* + K_p_)] e^(−kp *lag*)^. A rumen particulate outflow (K_p_) value of 3.0%/h, corresponding to ruminants at moderate intake levels [28], was utilized to estimate DMED values.

The amount of VFA in the bottles’ content after 24 h of incubation was corrected for the amount of VFA produced in the blanks. The proportion of minor VFA was calculated as the sum of isobutyrate, isovalerate and valerate.

Within each incubation run, the values measured in the two bottles incubated for each sample at each incubation time (144 or 24 h) were averaged before statistical analysis, resulting in three replicates per sample. The PROC MIXED of SAS [25] was used for all statistical analyses. In Experiment 1, data were analyzed as a mixed model which included the fruit (avocado or mango), the fraction (either peels or the PP mixture) and their interaction as fixed effects and the inoculum (incubation run) as a random effect. In Experiment 2, data were also analyzed as a mixed model in which the type of MB (AMB or MMB), the diet (MB alone or with alfalfa hay) and their interaction were considered as fixed effects and the effect of inoculum was random. Statistical significance was set at *p* < 0.05, and *p* values ≥ 0.05 and ≤0.10 were considered as trends.

## 3. Results and Discussion

### 3.1. Experiment 1. Chemical Composition and In Vitro Rumen Fermentation of Avocado and Mango Fruit Wastes

The chemical composition of avocado and mango wastes and of the two feeds used as reference (wheat middlings and corn grains) is shown in Table 2. As indicated previously, the proportions of pulp and peels in the PP mixtures represented their relative percentages in the fruits (on fresh weight basis), and were 81:19 for avocado and 65:35 for mango. Similar proportions of both fractions had been reported for avocado [4], but lower proportion of peels (7% to 24%) than in the present study were observed for mango [5,29], which can be due to the use of different peeling procedures.

The results from the present study agree with previously reported results [30] as the wastes from both fruits had low DM content (<250 g/kg fresh matter) and N (≤16.0 g/kg DM). Both DM and N levels were greater in avocado (averaged values for analyzed wastes: 240 g DM/kg and 15.9 g N/kg DM, respectively) than in mango samples (191 g DM/kg and 6.10 g N/kg DM). In accordance with previous results for mango [31,32], the peels contained more NDF and ADF than the PP mixture for both fruits. The peels from both fruits had similar NDF and ADF content, but the fiber from avocado peels was more lignified than that of mango peels (30.3 vs. 13.6 g lignin/100 g NDF). Negesse et al. [30] also reported similar content of both NDF and ADF in peels from avocado and mango, but the values reported by these authors were 2.2 times greater than those obtained in the present study. Chemical composition of avocado and mango fruits might vary depending on the fruit variety, cultivation conditions and ripeness, among other factors [4,33]. The high EE content in the avocado wastes agrees well with values from previously published studies [30,33], and explains the 1.7 times greater gross energy content of avocado wastes compared with mango wastes. Finally, the chemical composition of the two reference feeds was in the range of the values reported in the literature [34,35]. Both wheat middlings and corn grains are highly degradable in the rumen [34,35], but differ widely in their N and NDF content.

As shown in Table 3, there were no fruit × fraction interactions in either the gas production or the fermentation parameters, except for NH_3_-N concentrations (*p* = 0.011) and CH_4_ production (*p* = 0.039), although trends (*p* ≤ 0.092) were observed for DMED, TDMD, minor VFA and CH_4_/VFA ratio. Both PGP and AGPR were greater (*p* ≤ 0.002) for mango than for avocado. The gas production technique is based on the positive relationship between the amount of gas produced and that of organic matter fermented [36], and the greater gas production for mango compared with avocado would indicate that mango wastes were fermented at a greater extent. Mango wastes are reported to be rich in easily fermentable and degradable compounds [37] whose fermentation contributes significantly to gas production. In fact, the amount of NSC in mango reached 810 and 828 g/kg DM for peels and the PP mixture, respectively. The lack of *lag* time for any waste is indicative of a rapid start of fermentation.

As previously discussed by Marcos et al. [38], the gas production of high-EE samples should be interpreted with caution because ruminal fermentation of fat generates no gas, and fat can even reduce gas production due to the decreased CH_4_ [39]. In fact, CH_4_ production was lower (*p* < 0.001) for avocado than for mango samples, possibly due to the high EE content in avocado. The lack of differences (*p* = 0.513) between fruits in the CH_4_/VFA ratio suggests that the lower CH_4_ production observed for avocado compared to mango was due to the reduced fermentation of avocado, possibly caused by its high EE content. Unsaturated free fatty acids are toxic to fibrolytic bacteria [40] and avocado fat is rich in unsaturated fatty acids, usually accounting for more than 85% of total fatty acids [10,41].

The trend for lower (*p* ≤ 0.073) values of DMED and TDMD for avocado (548 and 808 g/kg DM, respectively; averaged values for peels and PP mixture) compared to mango (609 and 889 g/kg DM) supports the differences between both fruits observed in gas production parameters. Skenjana et al. [42] reported similar DMED values (529 g/kg DM) for avocado meal estimated for a rumen particulate outflow of 0.05/h. In contrast, Eliyahu et al. [7] and Negesse et al. [30] reported lower in vitro OM digestibility values for avocado pulp (300 g/kg) and peels (365 g/kg), which were attributed to the high EE and lignin content in the avocado samples. Discrepancies among studies might be related to differences in the chemical composition of the wastes, as the avocado peels from the study of Negesse et al. [30] had greater NDF and lignin content (261 and 121 g/kg DM, respectively) than the samples used in our study (≤115 and 34.8 g/kg DM, respectively). Our DMED values for mango wastes agree well with those of Sanon et al. [43], who reported a value of 610 g/kg for DM digestibility of mango peels. In contrast, Negesse et al. [30] reported a greater value (706 g/kg) for OM digestibility of mango peels estimated from an equation involving gas production and chemical composition. The high TDMD values obtained for mango wastes in our study (≥882 g/kg) are in accordance with previously reported values ranging from 840 to 970 g/kg DM [44,45].

The lower (*p* < 0.001) pH of mango compared with avocado samples agrees well with the greater (*p* < 0.001) total VFA production of mango wastes, because pH declines as VFA are produced and accumulated into the bottles. There were also differences between fruits in the VFA profile, and mango produced less (*p* = 0.007) acetate and more (*p* = 0.017) butyrate proportions than avocado wastes, and tended to greater (*p* = 0.092) propionate proportions than avocado. As a consequence, the acetate/propionate ratio was lower (*p* = 0.043) for mango, indicating a more efficient fermentation [46]. Differences in VFA profile are most likely related to the different carbohydrates’ composition in each fruit. The greater (*p* = 0.001) NH_3_-N concentrations of avocado compared with mango wastes are in accordance with the 2.6 times greater N content of avocado. As discussed by de Evan et al. [47], in vitro NH_3_-N concentrations are difficult to interpret, as they reflect the balance between the NH_3_-N produced by protein degradation and the NH_3_-N captured by the ruminal microorganisms for microbial protein synthesis. The high fermentation of mango wastes probably stimulated microbial growth, resulting in high NH_3_-N capture by the microorganisms and low NH_3_-N concentrations, which were below the level of 5 mg of NH_3_-N/100 mL recommended for optimal growth of ruminal microorganisms in vitro [48]. These results indicate that supplementation of mango wastes with N sources might improve their ruminal fermentation.

There were differences between wastes (peels and PP mixture) in some of the parameters analyzed. The greater (*p* ≤ 0.033) *c* and DMED values observed for the PP mixture compared to the peels indicate that pulp was fermented faster than peels for both fruits, as previously reported [49]. In contrast, the extent of ruminal degradation of both wastes was similar, as indicated by the lack of differences in the PGP and TDMD. The VFA profile was similar for both wastes excepting that the proportion of minor VFA was greater (*p* = 0.019) for the PP mixture than for the peels, with the difference greater for avocado than for mango (2.5 and 1.3 times greater, respectively). The greater proportion of minor VFA in the PP mixture of avocado compared with avocado peels is consistent with the greater NH_3_-N concentrations observed for the PP mixture, as both minor VFA and NH_3_-N are final products of protein degradation in the rumen, with isobutyrate and isovalerate being breakdown products of valine and leucine, respectively [45]. Because both the peels and the PP mixture had similar N content, these results might indicate that the protein of the PP mixture was more degradable than peel protein. This could be related to the presence of lower amounts of tannins in the peels than in the PP mixture [49], as tannins can form insoluble complexes with proteins and reduce their degradation [50]. Unfortunately, tannins content was not analyzed in the present study.

### 3.2. Experiment 2. In Vitro Fermentation of Multi-Nutrient Blocks (MB) Including Avocado and Mango Fruit Wastes

Table 4 shows the chemical composition of the feeds and diets used in Experiment 2. Dry matter content of both MB was high (≥905 g/kg). Although the MB were formulated to have similar N and fiber content, N, NDF and ADF levels were slightly greater in MMB compared with AMB. The more marked difference between the two MB types was in EE content, which was 1.8 times greater for AMB than for MMB; however, the fat level in both MB was below the maximal level of 60 g/kg diet DM generally recommended to avoid negative effects on fiber digestibility [34]. The two mixed diets including the MB (AMB and MMB) had similar chemical composition, with the exception of EE content, that was 1.4 times greater for the diet containing AMB than for that including MMB.

Table 5 shows the parameters of gas production kinetics and the main fermentation parameters for both types of MB, the two mixed diets and the alfalfa hay included in the mixed diets. There were no interactions (*p* ≥ 0.122) between the MB type and the fermentation substrate (MB alone or in mixed diets including alfalfa hay) in any of the parameters measured, indicating that the effect of mixing the MB with alfalfa hay on fermentation parameters was similar for both AMB and MMB. The only difference between MB in the gas kinetics parameters was detected in the PGP, which was greater (*p* = 0.010) for AMB than for MMB. However, the difference was low (105 and 99 mL gas/g DM incubated for avocado and mango, respectively; averaged values for MB incubated alone and mixed diets), and probably not relevant in practice. These results indicate that avocado fat had no negative effect on the in vitro fermentation of AMB when used in the given levels.

Compared with AMB, the fermentation of MMB tended to show greater (*p* = 0.096) pH values, which is consistent with the trend of lower VFA production (*p* = 0.054; 1.87 and 1.77 mmol/400 mg of DM) for AMB and MMB, respectively; averaged values for MB and mixed diets). Both MB differed in the VFA profile, as the fermentation of MMB resulted in greater (*p* ≤ 0.022) acetate proportions and acetate:propionate ratio, as well as in lower (*p* = 0.032) propionate proportion, than AMB fermentation. In contrast, NH_3_-N concentrations, CH_4_ production and CH_4_/total VFA ratio were similar for both MBs. The observed differences between MB in VFA production and profile are due not only to the characteristics of the PP mixture included in each MB, but also to the use of variable proportions of other feed ingredients in the MB. The proportions of the PP mixture from avocado and mango in the MB were different due to the high EE content of the avocado PP mixture, which precluded its inclusion at greater levels.

The changes in gas production and fermentation parameters observed when MB were included in mixed diets are in accordance with the fermentation pattern of alfalfa hay (Table 5). Compared with the fermentation of MB as the only substrate, their 1:1 mixture with alfalfa hay resulted in lower PGP (*p* = 0.018) and tended to lower TDMD (*p* = 0.058), which is consistent with the reduced values of these parameters observed for alfalfa hay compared with MB. In contrast, mixed diets had greater *c*, AGPR and DMED values compared with MB, as a result of the greater values observed for alfalfa hay. The larger values of VFA production, acetate proportion and acetate:propionate ratio measured for the mixed diets compared to MB are in accordance with the high rumen fermentability of the alfalfa hay used in this study, as indicated by its high values of *c*, AGPR and DMED values, low NDF proportion and high N content.

## 4. Conclusions

Both peels and pulp from avocado and mango had high-moisture content, but fiber content was greater in the peels than in pulp for both fruits. Mango fruit wastes were rich in non-structural carbohydrates, whereas those from avocado had high fat content, which reduced their ruminal fermentability. The inclusion of a pulp:peels mixture of either avocado or mango replacing conventional feeds in multi-nutrient blocks resulted in normal in vitro ruminal fermentation patterns, and therefore it might be a viable solution to feed ruminants and reduce the environmental impact of these wastes and animal feeding costs. Moreover, the inclusion of the multi-nutrient blocks in mixed diets with alfalfa hay as forage increased the fermentation rate of the diet. Additional studies are needed to assess the acceptance of the studied multi-nutrient blocks by ruminants and their stability over long-time storage periods.

## Figures and Tables

**Table 1:** Ingredient composition (g/kg fresh matter) of multi-nutrient blocks including a mixture of pulp and peels either from avocado tested in Experiment 2 ^1^.

Ingredient	AMB ^2^	MMB ^3^
Avocado pulp and peels	150	-
Mango pulp and peels	-	290
Sunflower meal	250	258
Wheat bran	221	200
Corn	220	100
Barley straw	80.0	90.0
Beet molasses	49.5	21.0
Quicklime	20.0	20.0
Urea	4.1	4.0
Palm soap	0.4	12.0
Vitamin–mineral mixture	5.0	5.0

^1^ The pulp:peels ratio (weight basis) in the mixture was 81:19 for avocado and 65:35 for mango; ^2^ AMB: multinutrient blocks including a mixture of pulp and peels from avocado; ^3^ MMB: multinutrient blocks including a mixture of pulp and peels from mango.

**Table 2:** Chemical composition (g/kg dry matter (DM) unless otherwise stated) of the avocado and mango wastes, and conventional feeds tested in Experiment 1.

Sample	DM (g/kg Fresh Matter)	Organic Matter (OM)	Nitrogen (N)	Neutral Detergent Fiber (NDF)	Acid Detergent Fiber (ADF)	Acid Detergent Lignin	Ether Extract (EE)	Lignification ^1^	Non-Structural Carbohydrates (NSC) ^2^	**Gross Energy** **(MJ/kg)**
Avocado and mango wastes ^3^										
Avocado peels	236	981	15.8	115	104	34.8	589	30.3	182	30.5
Avocado pulp:peels mixture	244	982	16.0	89.8	63.1	3.97	638	4.41	184	30.9
Mango peels	208	974	5.13	118	95.1	16.0	13.9	13.6	810	18.0
Mango pulp:peels mixture	174	975	7.07	95.5	63.5	11.3	7.34	11.8	828	17.5
Conventional feeds										
Corn grains	881	988	15.0	154	30.4	3.28	41.9	2.13	699	18.9
Wheat middlings	886	964	28.3	372	111	23.7	37.8	6.37	377	19.4

^1^ Calculated as [(lignin/NDF) × 100]; ^2^ Calculated as (OM − (N × 6.25) − NDF − EE); ^3^ The pulp:peels ratio (weight basis) in the mixture was 81:19 for avocado and 65:35 for mango.

**Table 3:** Gas production parameters, dry matter (DM) effective degradability (DMED) and true DM digestibility (TDMD) in 144 h incubations and fermentation parameters in 24 h incubations of avocado and mango wastes (either peels or a pulp:peels mixture) and two reference feeds (corn grains and wheat middling) with buffered ruminal fluid from goats (Experiment 1).

Parameter	Avocado		Mango		SEM ^1^	*p* =			Corn Grains	Wheat Middlings
	Peels	Pulp:Peels (81:19 *w*/*w*)	Peels	Pulp:Peels (65:35 *w*/*w*)		Fruit	Fraction	Fruit × Fraction		
Gas production parameters ^2^										
PGP (mL)	60.7	56.5	156	153	2.30	<0.001	0.215	0.809	157	123
*c* (per h)	5.18	7.56	6.04	7.21	0.474	0.628	0.033	0.292	4.32	8.12
*lag* (h)	0.00	0.00	0.00	0.00	-	-	-	-	1.62	0.00
AGPR (mL/h)	2.27	3.08	6.77	7.91	0.464	0.002	0.126	0.746	4.46	7.20
DMED (g/kg DM) ^3^	470	626	598	619	21.3	0.065	0.026	0.051	50.6	56.8
TDMD (g/kg DM) ^4^	742	874	895	882	29.8	0.073	0.140	0.092	919	780
Fermentation parameters										
pH	6.79	6.80	6.45	6.42	0.031	<0.001	0.841	0.558	6.56	6.68
Total volatile fatty acids (VFA; mmol/vial)	1.20	1.20	2.49	2.76	0.111	<0.001	0.317	0.327	2.33	2.21
Individual VFA (mol/100 mol)										
Acetate (Ac)	69.1	63.4	58.3	57.2	1.30	0.007	0.078	0.181	52.1	58.8
Propionate (Pr)	24.0	26.5	29.3	30.4	1.88	0.092	0.403	0.740	27.3	27.4
Butyrate	7.12	9.05	11.9	11.8	0.778	0.017	0.328	0.281	18.8	11.2
Minor VFA ^5^	0.431	1.06	0.513	0.649	0.0826	0.138	0.019	0.058	1.80	2.53
Acetate/Propionate (mol/mol)	2.90	2.39	2.01	1.89	0.207	0.043	0.224	0.422	1.96	2.17
NH_3_-N (mg/100 mL)	6.58	14.6	1.40	0.97	0.746	0.001	0.015	0.011	1.75	13.5
CH_4_ (ml/g DM incubated)	30.7	31.1	68.2	61.5	1.01	<0.001	0.052	0.039	67.6	57.6
CH_4_/VFA (mL/mmol)	10.2	10.4	11.0	8.95	0.42	0.513	0.105	0.083	11.6	10.4

^1^ SEM: standard error of the mean; ^2^ PGP: potential gas production; *c*: fractional rate of gas production; *lag*: delay until starting gas production; AGPR: average gas production rate; ^3^ DMED: Dry matter effective degradability for a rumen particulate outflow of 3.0%/h; ^4^ True dry matter degradability calculated according to Van Soest et al. [20]; ^5^ Sum of isobutyrate, isovalerate and valerate.

**Table 4:** Chemical composition (g/kg dry matter (DM) unless otherwise stated) of multi-nutrient blocks containing avocado (AMB) and mango (MMB) wastes, mixed diets composed of 1:1 alfalfa hay and multi-nutrient blocks, and alfalfa hay tested in Experiment 2.

Sample	DM (g/kg Fresh Matter)	Organic Matter (OM)	Nitrogen (N)	Neutral Detergent Fiber (NDF)	Acid Detergent Fiber (ADF)	Acid Detergent Lignin	Ether Extract (EE)	Lignification ^1^	Non-structural Carbohydrates (NSC) ^2^	**Gross Energy** **(MJ/kg)**
Multi-nutrient blocks and feed mixtures										
AMB ^3^	915	921	32.2	413	246	94.3	43.5	22.8	263	18.7
MMB ^4^	905	912	35.0	454	289	103	24.3	22.7	215	18.3
AMB: alfalfa hay	907	903	32.2	434	302	92.5	33.3	21.3	235	18.1
MMB: alfalfa hay	925	898	33.6	457	325	96.2	24.1	21.1	207	17.9
Other feeds										
Alfalfa hay	889	884	32.1	458	360	89.4	23.7	19.5	202	17.5

^1^ Calculated as [(lignin/NDF) × 100]; ^2^ Calculated as (OM − (N × 6.25) − NDF − EE); ^3^ AMB: multi-nutrient block containing a 81:19 (*w*/*w*) mixture of avocado pulp and peels; ^4^ MMB: multi-nutrient block containing a 65:35 (*w*/*w*) mixture of mango pulp and peels. Feed ingredient composition of AMB and MMB is given in Table 1.

**Table 5:** Gas production parameters, dry matter effective degradability (DMED) and true dry matter digestibility (TDMD) in 144 h incubations and fermentation parameters in 24 h incubations of multi-nutrient blocks containing avocado (AMB) or mango (MMB) wastes (pulp:peels) incubated either alone or mixed 1:1 with alfalfa hay with buffered ruminal fluid from goats (Experiment 2).

Parameter	AMB		MMB		SEM ^1^	*p* =			Alfalfa Hay
	Alone	Mixed Diet	Alone	Mixed Diet		MB ^2^	Fermentation Substrate	MB × Fermentation Substrate	
Gas production parameters ^3^									
PGP (mL)	108	101	99.8	97.2	1.02	0.010	0.018	0.122	91.0
*c* (per h)	4.76	6.29	4.68	6.43	0.110	0.788	<0.001	0.384	7.21
*lag* (h)	0	0	0	0	-	-	-	-	0.00
AGPR (mL/h)	3.71	4.58	3.37	4.51	0.101	0.131	0.002	0.279	4.73
DMED (g/kg DM) ^4^	443	483	450	474	8.2	0.952	0.030	0.413	486
TDMD (g/kg DM) ^5^	723	713	739	696	8.8	0.942	0.058	0.154	688
Fermentation parameters									
pH	6.76	6.80	6.82	6.85	0.024	0.096	0.215	0.775	6.79
Total volatile fatty acids (VFA; mmol/400 mg DM)	1.83	1.90	1.70	1.84	0.030	0.054	0.042	0.301	1.88
Individual VFA (mol/100 mol)									
Acetate (Ac)	60.4	65.0	62.2	66.3	0.31	0.015	0.001	0.451	68.9
Propionate (Pr)	27.7	24.2	25.5	22.9	0.47	0.032	0.008	0.386	22.2
Butyrate	10.0	7.96	10.2	7.87	0.344	0.904	0.008	0.721	5.37
Minor VFA ^6^	1.73	2.82	2.12	2.93	0.090	0.069	0.002	0.218	3.53
Acetate/Propionate (mol/mol)	2.18	2.68	2.45	2.89	0.055	0.022	0.003	0.623	3.11
NH_3_-N (mg/100 mL)	14.9	15.3	17.7	17.3	1.05	0.104	0.998	0.729	18.8
CH_4_ (ml/g dry matter incubated)	50.7	52.0	47.8	49.1	2.64	0.356	0.656	0.995	47.5
CH_4_/VFA (mL/mmol)	11.1	11.0	11.3	10.7	0.62	0.911	0.616	0.757	10.1

^1^ SEM: standard error of the mean; ^2^ MB: multi-nutrient block; ^3^ PGP: potential gas production; *c*: fractional rate of gas production; *lag*: delay until starting gas production; AGPR: average gas production rate; ^4^ DMED: Dry matter effective degradability for a rumen particulate outflow of 3.0%/h; ^5^ True dry matter degradability calculated according Van Soest et al. [20]; ^6^ Sum of isobutyrate, isovalerate and valerate.
